# An Electrically and Thermally Erasable Liquid Crystal Film Containing NIR Absorbent Carbon Nanotube

**DOI:** 10.3390/molecules27020562

**Published:** 2022-01-17

**Authors:** Zongcheng Miao, Dong Wang

**Affiliations:** 1 School of Artificial Intelligence, Optics and Electronics (iOPEN), Northwestern Polytechnical University, Xi’an 710072, China; miaozongcheng@nwpu.edu.cn; 2 School of Materials Science and Engineering, University of Science and Technology Beijing, Beijing 100083, China

**Keywords:** liquid crystal, NIR absorbent, carbon nanotube, phase transformation

## Abstract

Carbon nanotubes (CNTs) coated by a poly(vinylpyrrolidone) (PVP) layer were doped in bistable cholesteric liquid crystal (ChLC) film to provide electric, thermal, or optical erasability controllable films. The CNT/PVP formed a compatible NIR-absorbing film that can generate heat to switch ChLC film from a planar texture to a focal conic texture. The appropriate content of CNT/PVP is provided to achieve a fast thermal response, satisfactory dispersion, and clear display brightness. The ChLC film containing CNT/PVP @ 0.8 (wt.%) saves 51% time at thermal erasing, compared to the ChLC mixture without NIR absorbent. The hybrid organic–inorganic bistable ChLC material reported here extends and offers new applications of ChLC writing tablets.

## 1. Introduction

Cholesteric liquid crystal (ChLC)-based reflex displays are being widely applied in the electronic paper area, owing to paper-like reflectance, flexibility, bistability, and energy efficiency [1,2,3,4,5,6,7,8,9,10]. ChLCs are used extensively in various creations of flexible displays, mainly in reflective and flexible displays detection [11,12,13,14,15,16,17,18,19,20,21,22,23]. Schneider reported a reflective bistable ChLC containing a malleable touch-sensitive writing tablet where ChLC was sealed between two polyethylene terephthalate substrates, and both substrates were coated with conductive polymers [24]. The film, based on a polymer-dispersed ChLC developed by Yang, had two steady conditions at ground state, i.e., reflective planar and non-reflective focal conic texture. To detect what had been written, Lee studied a pressure-sensitive cholesteric display with an algorithm [25]. The ChLC writing board has achieved its repetitive properties based on bistability of structures in thin ChLC layers [1,5,26,27,28]. When pressure is applied on the surface of the writing board, ChLC orientation transforms the focal conic texture into a planar reflective texture, and the tablet is displayed. Then, an AC electric field is applied on the writing board, ChLC orientation transforms the planar reflective texture into focal conic texture, the incident light does not reflect, and hence the display image is erased. Nowadays, ChLC films such as smart windows can achieve both thermal and UV responses [29]. The most important challenge for the ChLC handwriting tablet is that users hope to clear part of the writing content.

To solve this problem, we introduce near-infrared (NIR) absorbent nanoparticles into the existing LC handwriting board. In this study, CNTs with strong light absorption capacity in a wide waveband of 800–1500 nm act as the NIR absorbent. CNTs have been previously utilized in many scientific areas, such as photothermal therapy and pharmaceutical analysis [30,31,32,33,34,35].

Here, a CNT is coated with PVP to create an interface between the CNT and LC mixtures, increase their compatibility, and render CNT dispersion in LC mixtures. A CNT/PVP acting as NIR absorbent exhibits high NIR absorption efficiency and produces a great amount of energy, which partially heats the LC mixture to its clearing point. To verify its partial phase transformation ability, we have designed a thermal erasing experiment.

## 2. Results and Discussion

### 2.1. Morphological Observation

**Figure 1:**
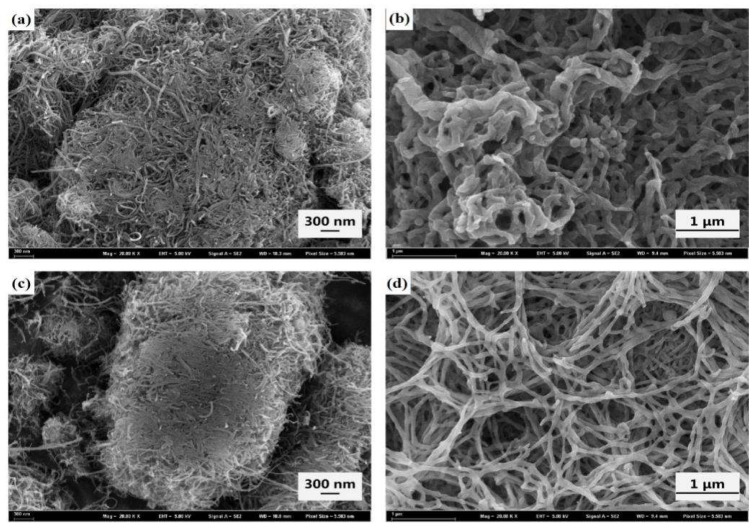
SEM images of the morphology of the CNT: (**a**) unmodified CNT and (**c**) modified CNT/PVP. SEM photographs of the surface morphology of the LC cells containing (**b**) unmodified CNT and (**d**) modified CNT/PVP.

The surface morphology and size of the neat CNT and CNT/PVP were observed by SEM (Figure 1). The difference in particle dispersion was also observed using SEM. The SEM image of the uncoated CNT is shown in Figure 1a. Spontaneous aggregation of CNTs was observed after being introduced in the LC mixture (Figure 1b). Due to CNT aggregation loss in the transparency of the visible region, poor NIR absorbing performance became prominent. The morphology of CNT/PVP is shown in Figure 1c. CNT/PVP had a similar size as unmodified CNT. Due to the coating of PVP, the surface of CNT/PVP presents a higher brightness in the SEM photograph. As shown in Figure 1d, CNT/PVP was well dispersed within the polymer structure. After PVP coating, the aggregation of CNTs was reduced. Coating the CNT surface with PVP reduced the incorporation percentage of CNT into the LC mixture due to the presence of amide in the PVP layer. These amides interfere with CNTs and a highly compatible mixture containing LCs and polymerizable monomers.

**Figure 2:**
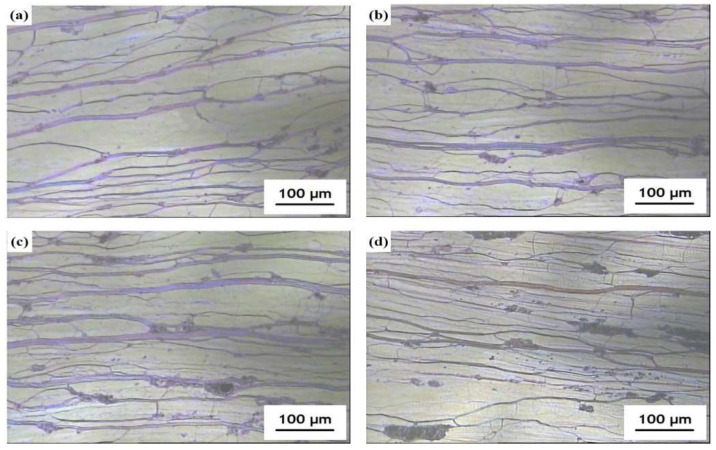
POM images of LC samples doped with various modified CNT/PVP concentrations: (**a**) 0.2 wt.%, (**b**) 0.5 wt.%, (**c**) 0.8 wt.%, and (**d**) 1.0 wt.%.

The POM photographs of mixtures **5**–**8** after UV curing are shown in Figure 2. All samples show well-aligned planar textures of ChLC, which is also the reflective state of ChLC. It was observed that the CNT/PVP aggregation size changed due to the concentration of particles. As shown in Figure 2a,b, less CNT/PVP aggregation was prominent, while in Figure 2c,d, large-scale aggregation CNTs can be seen in the LC mixture. Furthermore, the violet areas in the POM photographs mean a hairspring texture related to the LC defect. The gray areas mean that CNTs or CNT/PVP aggregate in the LC. It can be seen that LC mixtures containing a relatively high concentration of CNT/PVP had poor uniformity, and more flaws were prominent in LC textures.

We make a preliminary conclusion to proceed towards the next experiments with an evenly dispersed distribution of CNTs. It is concluded from the POM images that CNT/PVP @ 1.0 (wt.%) was found to achieve good dispersion of CNT/PVP.

### 2.2. Optical Properties

**Figure 3:**
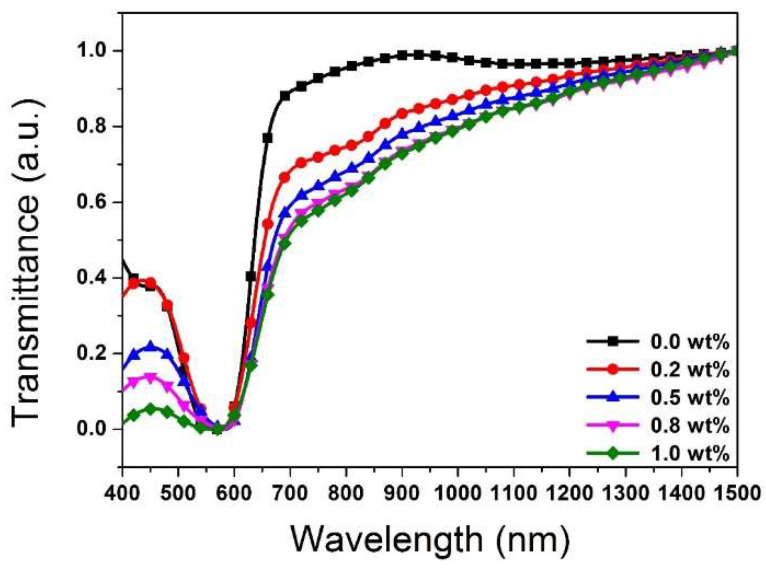
UV transmittance spectra of LC mixtures with different concentrations of CNT/PVP (samples **0**, **5**, **6**, **7**, and **8**).

The transmittance spectra of LC mixtures (samples **0**, **5**, **6**, **7**, and **8**) after UV irradiation are shown in Figure 3. The light transmittances of the samples were measured using a UV/vis spectrophotometer. The transmittance of a blank cell was normalized as 1.0. The central reflection wavelength of all samples is close to 560 nm. It is clear that CNT/PVP slightly distressed the transmittance of LC mixtures at the visible band. The transmittance of the LC mixture changed due to CNT/PVP, and the LC mixture with a higher concentration of CNT/PVP showed a better NIR absorbing performance. The transmittance also changed from 700 to 1500 nm due to CNT/PVP. At a wavelength of 808 nm, the regularity of the NIR absorption of different samples was obvious (Figure 3). Samples **6**, **7**, and **8** with CNT/PVP concentrations of 0.5, 0.8, and 1.0 wt.% demonstrated excellent NIR absorbing capacity; however, samples 0 and 5 showed poor NIR absorbing capacity due to non-uniform dispersion of CNTs or lower CNT content.

Keeping in mind the dispersion and absorption performance, sample **7**, with a CNT/PVP concentration of 0.8 (wt.%), was chosen for the thermal erasing experiment to verify the actual effect of CNT/PVP.

### 2.3. Heating Erasing Properties

**Figure 4:**
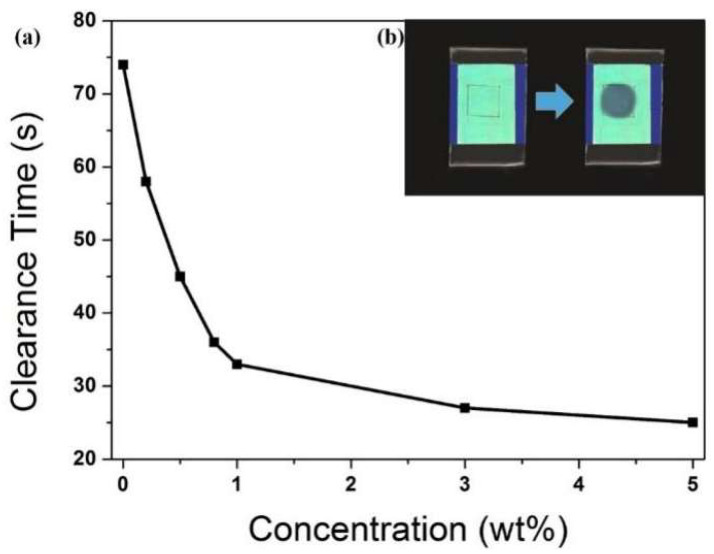
(**a**) The clearance time of different LC samples in the thermal erasing experiment. (**b**) The photographs of sample **7** before and after the thermal erasing experiment.

The thermal erasing experiment was designed to find the regularity of the thermal erasing property of CNT/PVP. Photographs of sample **7** before and after the thermal erasing experiment are shown in Figure 4a. The marked area of the LC mixture was irradiated and cleared using a NIR lamp (980 nm) at a light intensity of 30 mW/cm^−2^. CNT/PVP acts as a radiator and converts the absorbed near-infrared energy to heat; thus, it initiates the transition of the planar state to the liquid phase. The time consumed to clear the 1 cm^2^ area of LC mixtures was recorded, and the mean value was computed. The results of the thermal erasing experiment are shown in Figure 4b. LC film with a higher concentration of CNT/PVP took less time to complete the phase transformation. The clearance time decreased dramatically from sample 0 to 8 (wt.%) (42 s were saved). When the concentration of CNT/PVP in the sample increased more than sample 8, the clearance time did not improve further, leveling off to 32 s.

The inclusion of an appropriate concentration of CNT/PVP enhances the exothermic efficiency of NIR irradiation in the LC mixture without interfering with the reflection in the visible region. After taking dispersion, transparency, and NIR heating erasing time into account, sample **7** was found to be most suitable. Compared to LC film without CNT/PVP, sample 7 showed a fast thermal response, satisfactory dispersion, and clear display brightness.

**Figure 5:**
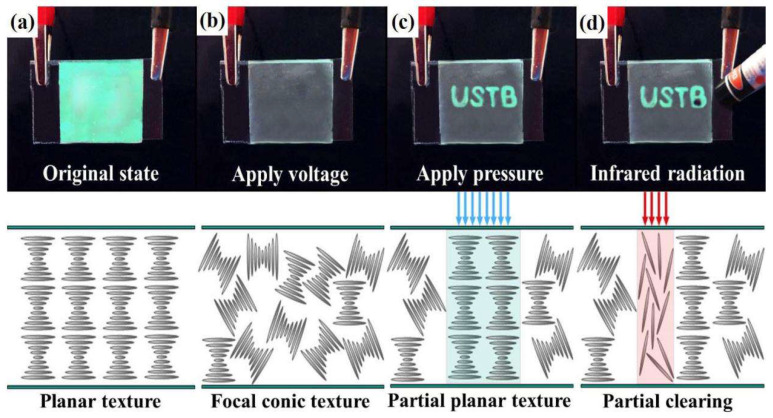
Four main stages of the LC cell: (**a**) reflective state with planar texture. (**b**) Applied voltage to reflective LC cell, the mixture would change into the focal conic texture. (**c**) When applying pressure to non-reflective samples, the stressed part would return to planar texture. (**d**) When irradiating the NIR light source at the pattern, the partial LC cell would be heated rapidly to the clearing point. The molecular arrangement of every stage was directly below the specific image of the stage.

Sample **7** (containing CNT/PVP @ 0.8 wt.%) was filled into ITO-PET cells to make a simple writing tablet model (Figure 5). In Figure 5a, the whole writing tablet displays bright green. When an electric field was applied to the cell, the LC orientation transformed from planar reflective texture into focal conic texture, the LC could not reflect the incident light, and the cell displayed high transparency (Figure 5b). The pressure-sensitive tablet exhibited the English letters “USTB” (Figure 5c). Pressure at the surface of the writing tablet was provided by a stylus, and then the LC optically responded to the pressure. The NIR light source was shone on the writing “USTB”, and the capital “B” was partially erased (Figure 5d).

The phase-transferring process can be explained from the perspective of the molecular arrangement of the LC mixture. As shown in Figure 5a, the writing tablet exhibited a well-aligned planar texture. At this time, helically twisted molecules of LC allow the light to pass through the cell, which is perpendicular to the substrate. The writing tablet was electrically switched to the focal conic state as shown in Figure 5b. The focal conic texture has a multi-domain structure where the helical axes are randomly arranged throughout the cell. The incident light does not reflect, and hence the writing is erased. As shown in Figure 5c, the writing tablet is pressure sensitive. The texture of ChLC transfers from the focal conic state to the planar reflective texture. The writing tablet was selectively erased by NIR light (Figure 5d). NIR light heats the LC mixture rapidly to the clearing point. At this stage, the heated LC mixture loses liquid crystallinity and turns to the liquid phase. From Figure 5a to Figure 5b, a voltage pulse is applied to transfer the phase of the ChLC mixture from planar texture to the focal conic state.

In this study, NIR absorbent was applied to transfer the reflective planar state to the liquid phase. The handwriting was partially erased (Figure 5c,d). A voltage pulse was applied to transfer the phase of the ChLC mixture from a planar texture to the focal conic state (Figure 5a,b).

**Figure 6:**
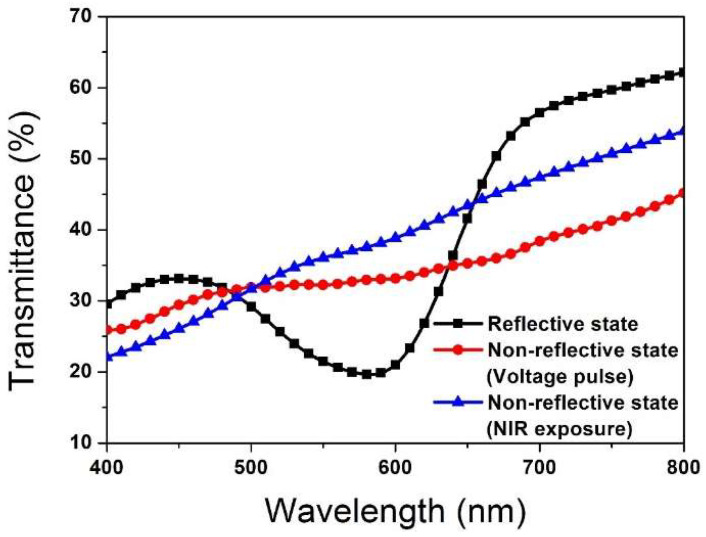
UV transmittance spectra of sample 7 at the reflective state and non-reflective state (induced by voltage pulse and NIR).

The sample erased by voltage showed moderate transparency from 400 to 800 nm. Figure 6 shows the transmission spectra (400–800 nm) of all three states. When applying pressure to non-reflective samples, the stressed part returns to planar texture. The central reflection wavelength of all samples is close to 560 nm. When aiming the NIR light source at the pattern, the partial LC cell is heated rapidly to the clearing point. It can be seen that, on non-reflective states, both the electrical erasing and NIR erasing show similar transmittance from 400 to 800 nm. The small distinction in transmittance has an apparent difference in operation, since they both looked nearly transparent on a dark background.

## 3. Experimental Work

### 3.1. Materials

**Scheme 1:**
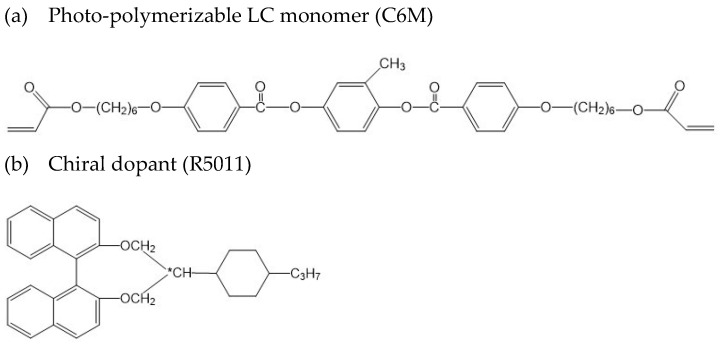
The chemical strusture of (**a**) C6M and (**b**) R5011.

CNTs were purchased from Jiangsu Xianfeng Nanomaterials CO., LTD. Nanjing, China, and the modifying agent poly(vinylpyrrolidone) (PVP) was purchased from Xilong Scientific Co., Ltd. Shantou, China. LC samples were designed to study the dispersion of CNTs and their heat-clearing ability. The ingredients used as nematic were as follows: LC (5CB, 4-cyano-4-pentylbiphenyl, no = 1.532, TN-1 = 35.5 °C, Shijiazhuang Chengzhi Yonghua Display Material Co., Ltd., Shijiazhuang, China), photo-polymerizable LC monomer, (C6M, synthesized in our lab), chiral dopants (R5011, Bayi Space Liquid Crystal Co., Ltd., Beijing, China), and a photo-initiator (IRG651, Energy Chemical Co., Ltd., Shanghai China). The chemical structural formulas of the main components are as follows (Scheme 1):

### 3.2. Modification of CNTs

The modification of CNTs is based on a previously reported procedure [36]. The whole procedure for the physical modification of CNTs is briefly described as follows.

**Figure 7:**
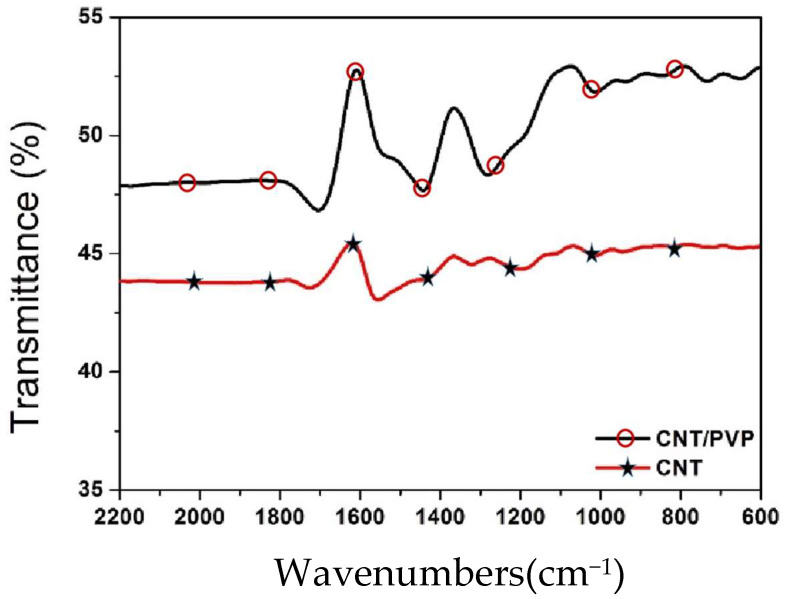
FTIR spectra of modified CNT/PVP.

Firstly, original CNT residues were added to distilled water, and homogenous dispersion was achieved by the ultrasonication process. Then, a small quantity of PVP aqueous solution was added, followed by continuous shaking for 24 h to allow complete absorption of PVP onto CNTs. After that, it was centrifuged and washed with distilled water following the dispersion of PVP-functionalized CNT (CNT/PVP) in polymeric syrup as well as other organic solvents such as ethanol and acetone in order to remove the residual moisture in the polymers completely. Fourier transform infrared (FTIR) spectra of modified CNT/PVP are shown in Figure 7. The peak of C=O stretching vibration at 1655 cm^−1^ was strengthened, and the peak of C-N stretching vibration appeared at 1290 cm^−1^ when comparing curves for CNTs and CNT/PVP, showing that the CNT and PVP were well combined.

### 3.3. Sample Preparation

Firstly, we mixed the C6M/chiral dopant/photo-initiator/LC. After that, this mixture was tested to ensure that the film reflected the wavelength with high brightness and contrast. The mass percentages of the components were as follows: C6M 3%, R5011 2.5%, IRG651 0.5%, and 5CB 94%. The LC mixture was shaken and mixed under ultrasonication and then stored in a high vacuum at 70 °C for 2 h to obtain a homogenous ChLC mixture. Different amounts of CNT/PVP powders and unmodified CNT powders were doped into the mixture at concentrations (wt.%) of 0, 0.2, 0.5, 0.8, and 1.0, respectively. The organic–inorganic composite mixture was blended by shaker and then evenly mixed under ultrasonication to obtain a homogenous ChLC–CNT mixture. Table 1 shows the composition of the samples.

In this study, the LC samples were injected into two different types of LC cells according to the requirement. LC samples were injected into traditional indium tin oxide (ITO) glass cells to observe morphological and optical characteristics and into poly(ethylene terephthalate) (PET)/ITO glass cells to study the thermal erasing performance. The PET/ITO glass cell consisted of a piece of ITO glass and a PET film (coated by ITO). The ITO glass acted as a hard substrate, while the PET film with a layer of ITO conductor was flexible. As partial pressure and thermal application require good flexibility, long-term stability, and wide temperature range functionality, the PET/ITO glass cell met the above-mentioned criteria for further observation and experimentation [37,38,39,40,41,42].

The LC mixture was injected into either cell with a 15.0 ± 1.0 μm gap under capillarity. For polymerization, the LC samples were irradiated for 15 min at room temperature using a UV lamp (4 mW/cm^2^, 365 nm). Then, samples were kept for further observations and experimentations.

### 3.4. Morphological Analysis

A diverging photosensitive microscope (POM, Olympus BX51, Tokyo, Japan) was used to observe the texture of LC samples. An SEM (scanning electron microscope) (ZEISS, EVO18, Oberkohen, Germany) was used to observe the structure of polymer linkages. To extract the LC molecules, all the prepared LC films were immersed in petroleum ether for 12 h. They were then dried to evaporate the solvent for 12 h. After that, all the samples are stammered with carbon and observed by SEM. The microstructure of the polymer network and the morphology of tiny CNT clumps in the polymer network were investigated.

### 3.5. Performance Analysis

For performance analysis, the phase transformation of LC mixtures was initiated using incident light of 880 nm and 500 mW/cm^2^. The NIR absorbing performance of the LC mixture was observed in ITO glass cells. A 1 cm^2^ square area was marked on the top glass of the ITO cell; then, the irradiation range of incident light was modelled at the center of the marked area. The time consumed to clear the 1 cm^2^ area of the LC mixture was recorded, and the mean value was computed.

## 4. Conclusions

In this article, an LC mixture with the NIR absorbent CNT/PVP was prepared to fabricate a thermally controlled NIR absorbing LC film. The well-dispersed CNT/PVP maximized the NIR absorbing capacity and demonstrated a higher thermal conversion rate under NIR exposure. The well-dispersed CNT/PVP generated energy more efficiently and initiated phase transformation efficiently, while heterogenous CNT/PVP film acted inefficiently, and the heat was dissipated, leading to a poor phase transformation ability. The appropriate content of CNT/PVP is provided in this study to achieve fast thermal response, satisfactory dispersion, and clear display brightness at the same time. The LC writing tablet presented in this article can be selectively erased by NIR light. NIR light heated the LC mixture rapidly to the clearing point, and the heated LC mixture lost liquid crystallinity and turned to liquid phase. It is believed that the display performance of the NIR absorbing LC film will be improved with further efforts. The LC writing tablet presented in this article would help to increase market penetration for ChLCDs.

## Figures and Tables

**Table 1:** The composition of the samples.

Samples	CNT wt.%	Samples	CNT–PVP wt.%
**0**	0.0	**0**	0.0
**1**	0.2	**5**	0.2
**2**	0.5	**6**	0.5
**3**	0.8	**7**	0.8
**4**	1.0	**8**	1.0

## Sample Availability

Samples of the compounds are not available from the authors.
